# Retinal response to systemic inflammation differs between sexes and neurons

**DOI:** 10.3389/fimmu.2024.1340013

**Published:** 2024-02-07

**Authors:** Kristy T. Rodríguez-Ramírez, María Norte-Muñoz, Fernando Lucas-Ruiz, Alejandro Gallego-Ortega, Francesco Calzaferri, David García-Bernal, Carlos M. Martínez, Caridad Galindo-Romero, Cristóbal de los Ríos, Manuel Vidal-Sanz, Marta Agudo-Barriuso

**Affiliations:** ^1^ Grupo de Investigación Oftalmología Experimental, Departamento de Oftalmología, Optometría, Otorrinolaringología y Anatomía Patológica, Facultad de Medicina, Universidad de Murcia, Instituto Murciano de Investigación Biosanitaria (IMIB), Murcia, Spain; ^2^ Instituto-Fundación Teófilo Hernando and Departamento de Farmacología, Facultad de Medicina, Universidad Autónoma de Madrid, Madrid, Spain; ^3^ Grupo de Trasplante Hematopoyético y Terapia Celular, Departamento de Bioquímica y Biología Molecular B e Inmunología, Facultad de Medicina, Universidad de Murcia, Instituto Murciano de Investigación Biosanitaria (IMIB), Murcia, Spain; ^4^ Plataforma de Patología, Instituto Murciano de Investigación Biosanitaria (IMIB), Murcia, Spain; ^5^ Departamento de Ciencias Básicas de la Salud, Universidad Rey Juan Carlos, Alcorcón, Spain

**Keywords:** male, female, lipopolysaccharide, inflammation, central nervous system, neuronal death, extrinsic apoptosis, inflammasome

## Abstract

**Background:**

Neurological dysfunction and glial activation are common in severe infections such as sepsis. There is a sexual dimorphism in the response to systemic inflammation in both patients and animal models, but there are few comparative studies. Here, we investigate the effect of systemic inflammation induced by intraperitoneal administration of lipopolysaccharide (LPS) on the retina of male and female mice and determine whether antagonism of the NLRP3 inflammasome and the extrinsic pathway of apoptosis have protective effects on the retina.

**Methods:**

A single intraperitoneal injection of LPS (5 mg/kg) was administered to two months old C57BL/6J male and female mice. Retinas were examined longitudinally *in vivo* using electroretinography and spectral domain optical coherence tomography. Retinal ganglion cell (RGC) survival and microglial activation were analysed in flat-mounts. Retinal extracts were used for flow cytometric analysis of CD45 and CD11b positive cells. Matched plasma and retinal levels of proinflammatory cytokines were measured by ELISA. Retinal function and RGC survival were assessed in animals treated with P2X7R and TNFR1 antagonists alone or in combination.

**Results:**

In LPS-treated animals of both sexes, there was transient retinal dysfunction, loss of vision-forming but not non-vision forming RGCs, retinal swelling, microglial activation, cell infiltration, and increases in TNF and IL-1β. Compared to females, males showed higher vision-forming RGC death, slower functional recovery, and overexpression of lymphotoxin alpha in their retinas. P2X7R and TNFR1 antagonism, alone or in combination, rescued vision-forming RGCs. P2X7R antagonism also rescued retinal function. Response to treatment was better in females than in males.

**Conclusions:**

Systemic LPS has neuronal and sex-specific adverse effects in the mouse retina, which are counteracted by targeting the NLRP3 inflammasome and the extrinsic pathway of apoptosis. Our results highlight the need to analyse males and females in preclinical studies of inflammatory diseases affecting the central nervous system

## 1 Introduction

Systemic inflammatory disorders, such as those caused by uncontrolled bacterial or viral infections, are associated with cognitive and memory impairment and exacerbation of neurocognitive diseases (1–6).

Sepsis is defined as life-threatening acute organ dysfunction secondary to bacterial infection (7). It is a complex and rapidly progressive medical problem in which several factors interact and determine the patient’s prognosis. It affects 19 million patients worldwide annually (8). Of these, half recover, one-third die within the next year, and one-sixth subsequently develop severe persistent neurological impairments (2, 3, 9). Clinical management of patients (10) does not include the treatment or prevention of neurological disorders, although it is an area of intense research (11).

Sepsis-induced neuronal dysfunction is thought to be caused by an exaggerated inflammatory response that, in the central nervous system (CNS), leads to blood-brain barrier dysfunction (12), neuroinflammation, oxidative stress, neurotransmitter imbalance (13, 14), decreased metabolism (15) and ultimately neuronal injury and death, the latter varying between brain areas (16, 17). These pathological processes are thought to be caused by activated macro- and microglial cells, and infiltrating peripheral immune cells that release pro-inflammatory cytokines [reviewed in (14, 18)].

There are several models of sepsis in rodents (19–21). One of the most widely used is based on the administration of the Gram-negative bacterial endotoxin lipopolysaccharide (LPS), which causes an increase in proinflammatory cytokines such as interleukin-1β (IL-1β) and tumour necrosis factor-alpha (TNF), microglial activation, and cognitive decline in rodents (22–25).

Although the mechanism underlying LPS-induced neuroinflammation and impairment is unknown, it has been reported that systemic LPS enters the CNS, albeit at low levels (26), and may therefore directly activate microglial cells. In addition, inflammatory caspases are directly activated by LPS through a non-canonical mechanism (27). In line with this, it has been shown that inhibition of the P2X7 receptor (P2X7R), which is activated by extracellular ATP and damage-associated molecular patterns (DAMPs) thereby promoting the assembling and activation of the NLRP3 inflammasome pathway, ameliorates cognitive decline in LPS-treated mice and reduces the levels of proinflammatory cytokines in the brain (28). Neuronal death caused by systemic LPS may be triggered as well through the activation of the extrinsic apoptosis pathway by TNF, the canonical ligand of the death receptor TNFR1 (tumour necrosis factor receptor 1a) (29).

Importantly, there is a sexual dimorphism in response to systemic inflammation in both patients and animal models (30–36). In fact, females usually have a better outcome. However, despite the increasing evidence of sexual dimorphisms in neurological and immunological systems, research into these differences in sepsis-associated neurodegeneration remains critically understudied (30).

The retina is the window to the brain, and offers several advantages compared with other CNS areas to study neurodegeneration and neuroprotection (37, 38). However, despite its advantages, little is known about how the retina responds to systemic inflammation, and no comprehensive studies comparing females and males have been reported.

Here, we compared the functional and anatomical changes in the retina in response to LPS in males and females. We focused on the ganglion cell layer, where we studied the fate of retinal ganglion cells (RGCs), the only afferent retinal neurons. RGCs consist of two functional subtypes, those implicated in vision-forming roles and those that elicit non-vision-related light responses. Vision-forming RGCs are the majority and express Brn3a, a transcription factor that is also a viability marker (Brn3a^+^RGCs) (39). Non-vision forming RGCs are detected by their melanopsin expression (m^+^RGCs), *i.e.* the chromophore that renders them intrinsically photosensitive (ipRGCs) (39, 40). ipRGCs are more resilient to several insults than Brn3a^+^RGCs (41, 42). Within this framework, we have performed longitudinal *in vivo* functional and anatomical analyses in septic mice. In post-mortem samples, we have assessed the survival of vision and non-vision forming RGCs, microglial dynamics and activation, and measured the levels of pro-inflammatory cytokines in matched plasma and retinal samples. Finally, we have tested the neuroprotective potential of P2X7R and TNFR1 antagonists, alone or in combination, to prevent neuronal damage associated with systemic inflammation.

## 2 Material and methods

### 2.1 Animal handling

All animal procedures were approved by the Institutional Animal Care and Use Committee of the University of Murcia (Murcia, Spain) and performed according to our institutional guidelines (approved protocol A13210201) and ARRIVE guidelines.

Two months old C57BL/6J male and female mice were obtained from the breeding colony of the University of Murcia or purchased from Envigo (Barcelona, Spain). Animals were kept at the University of Murcia animal housing facilities in temperature and light controlled rooms (12 h light/dark cycles) with food (pellet 12 mm, Teklad Global Diet^®^, Inōtiv, Mucedola, Milán, Italy) and water administered *ad libitum*.

### 2.2 Anaesthesia and euthanasia

Optical coherence tomography was conducted with general inhalational anesthesia utilizing 3% isoflurane (Abbott Laboratories, Abbott Park, IL) at a rate of 1.5 L/min oxygen, employing a calibrated precision vaporizer. For electroretinogram analyses, animals underwent anesthesia through an intraperitoneal injection of a combination of ketamine (60 mg/kg, Ketolar, Parke-Davies, S.L., Barcelona, Spain) and xylazine (10 mg/kg, Rompún, Bayer S.A., Barcelona, Spain). Following anesthesia, a protective ointment (Tobrex; Alcon S.A., Barcelona, Spain) was applied to the eyes to prevent corneal desiccation. The euthanasia process involved an intraperitoneal injection of a lethal dose of sodium pentobarbital (Dolethal, Vetoquinol; Especialidades Veterinarias, S.A., Alcobendas, Madrid, Spain).

### 2.3 Animal groups and experimental design

**Figure 1:**
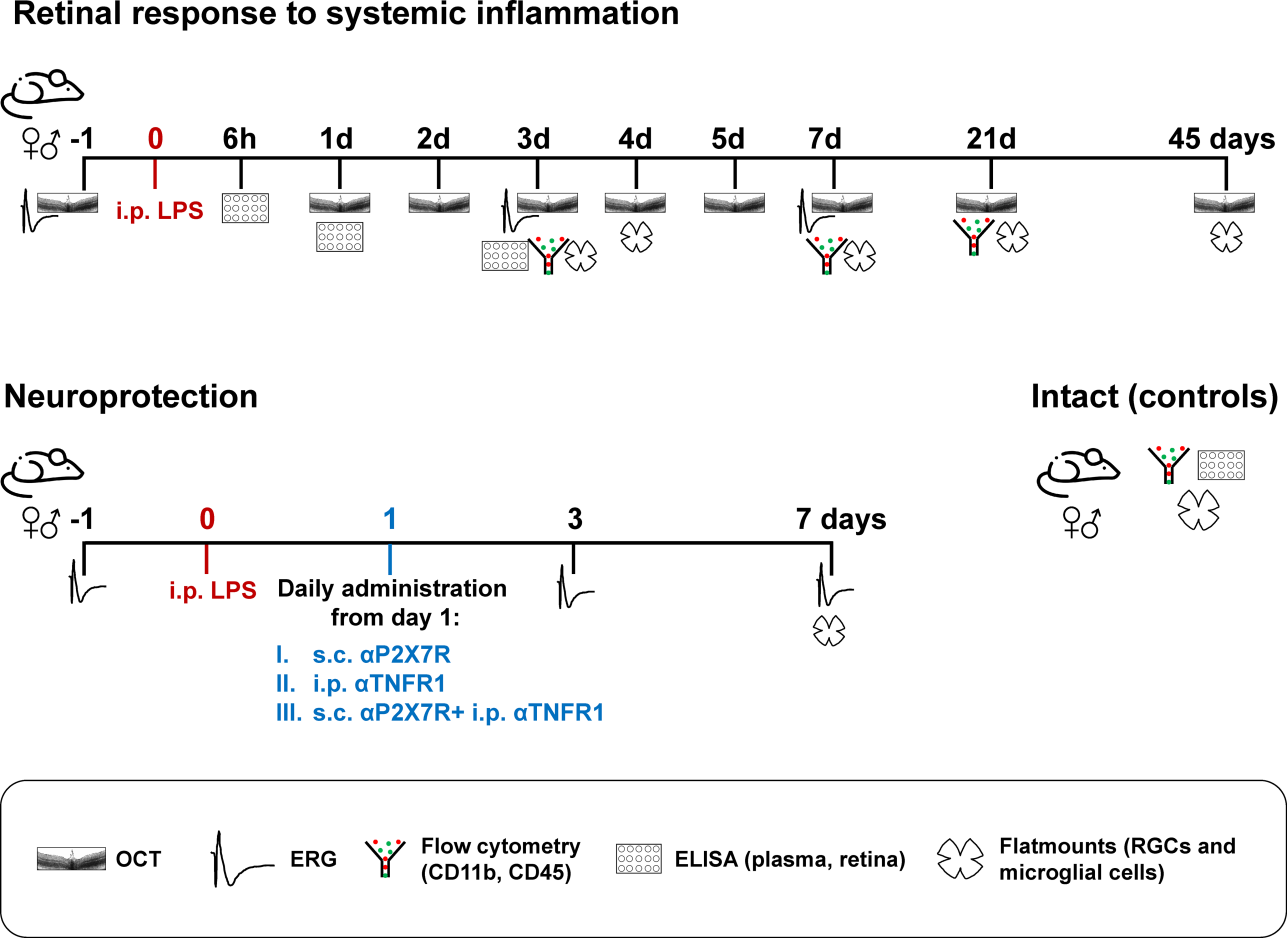
Experimental design. n=4-12 retinas or plasma/time/sex/analyses.

**Figure 1** summarises the experimental groups and analyses. *In vivo* functional and anatomical analyses (ERG and OCT) were performed longitudinally before (pre- or baseline) and after the procedures, and animals sacrificed at different time points for anatomical analyses on flat-mounts. New groups were done for cytometry and ELISA assays. Intact animals were used as controls for the total number of RGCs, morphology of resting microglia, flow cytometry, and cytokine profiling analyses. The number of samples per assay, sex and time point is shown in the scatter dot plots.

### 2.4 Intraperitoneal and subcutaneous injections

LPS [dissolved in saline. E. coli O111: B4, 437627 Millipore, Merck Life Science S.L.U. Madrid, Spain] and TNFR1 antagonist [12 mg/kg i.p. in 5% of DMSO-saline; R7050, Tocris Bioscience; Bio-Techne R&D Systems, Madrid, Spain], as previously published (43), were both injected intraperitoneally in a final volume of 200 µL.

P2X7R antagonist [ITH15004] was injected subcutaneously in a final volume of 100 µL of 10% DMSO/saline. ITH15004 (2-[6-chloro-9*H*-purin-9-yl)-1-(2,4-dichlorophenyl]ethan-1-one) is a non-nucleotide purine derivative that has shown selective P2X7 receptors blocking properties, and it has been synthesized accordingly to the method recently described (44).

For LPS and the P2X7R antagonist, we did a dose titration (see results) and the selected final concentrations were 5 mg/kg for LPS and 15 mg/kg for the P2X7R antagonist.

### 2.5 Electroretinography

The full-field electroretinogram (ERG) was conducted following previously published methods (45–47). Briefly, mice underwent 12 hours of dark adaptation, followed by anesthesia, and dilation of both eyes with a topical mydriatic (Tropicamide 1%; Alcon-Cusí, S.A., Barcelona, Spain). Scotopic and photopic responses were simultaneously recorded using Burian-Allen corneal bipolar electrodes. Methylcellulose (Methocel^®^ 2%; Novartis Laboratories CIBA Vision, Annonay, France) was applied between the cornea and electrodes to enhance signal conductivity. The reference electrode was positioned in the mouth, and a needle at the tail base served as the ground electrode. Scotopic responses reflecting retinal ganglion cell (RGC)-mediated activity were recorded with light flashes ranging from -4.4 log cd·s/m^2^, while rod-mediated responses were recorded at -2.5 log cd·s/m^2^. Mixed responses (a- and b-waves) were recorded at 0.5 log cd·s/m^2^. For cone-mediated responses, a flash of 0.5 log cd·s/m^2^ was applied on a 30 cd/m^2^ rod-saturated background. The electrical signals were digitized at 20 KHz using a Power Lab data acquisition board (AD Instruments, Chalgrove, UK), and standard ERG waves were analyzed in accordance with the International Society for Clinical Electrophysiology of Vision (ISCEV).

### 2.6 Spectral domain optical coherence tomography

Retinas were longitudinally examined using SD-OCT (Spectralis; Heidelberg Engineering, Heidelberg, Germany) adapted with a commercially available 78-D double aspheric fundus lens (Volk Optical, Inc., Mentor, OH, USA) positioned in front of the camera unit, as outlined in a previous publication (48). Following anesthesia, tropicamide eye drops (Tropicamide 1%; Alcon-Cusí, S.A. Barcelona, Spain) were administered to induce mydriasis in both eyes. Imaging was conducted with the proprietary software package (Eye Explorer, version 3.2.1.0; Heidelberg Engineering). A raster scan comprising 25 equally spaced horizontal B-scans was used to capture retinal images. Manual measurements of total, inner, and outer retinal thickness were taken near the optic nerve head (0.4 mm) and at a 1-mm distance from it, always within central sections spanning the optic disc. Subsequently, the software calculated the volume of the central retina, with manual alignment of inner and outer retinal limits in the 25 sections acquired per retina. Hyperreflective puncta in the vitreous were manually quantified in 3 central OCT sections/animal.

### 2.7 Tissue processing and immunodetection

Euthanized animals underwent transcardial perfusion with a 0.9% saline solution, followed by 4% paraformaldehyde in 0.1 M phosphate buffer solution. Flat-mounted retinas were prepared according to established procedures (49). Immunodetection procedures followed previous protocols (50). Flat-mounted retinas underwent triple immunodetection with mouse anti-Brn3a (1:500; MAB1585, Merck Millipore; Madrid, Spain) and rabbit anti-melanopsin (1:750; UF008 (AB-N39), Advanced Targeting Systems, Carlsbad, CA, USA) antibodies to quantify the total number of vision-forming and non-vision-forming retinal ganglion cells (RGCs), respectively. Additionally, guinea pig anti-Iba1 antibody was used to identify microglial cells or infiltrated macrophages (1:500; 234308, Synaptic Systems, Göttingen, Germany).

Secondary detection employed Alexa Fluor-labeled secondary antibodies (1:500; Molecular Probes; Thermo Fisher Scientific, Madrid, Spain). Retinal whole-mounts were mounted using anti-fading mounting media (H-1200, Vectashield^®^, Vector Laboratories Inc., Burlingame, CA, USA).

### 2.8 Image acquisition and analyses

Images were captured using a Leica DM6B epifluorescence microscope (Leica, Wetzlar, Germany). Retinal photomontages were constructed from individual square images of 500 µm² each. The total population of Brn3a^+^RGCs was automatically quantified following established procedures (49). m^+^RGCs were manually marked on the photomontage, and the markings were subsequently automatically quantified. The topographic distribution of Brn3a^+^RGCs and m^+^RGCs was evaluated through isodensity and neighborhood maps, respectively, using methods previously described (49, 51). Isodensity maps depict RGC density with a color scale ranging from 0-500 (purple) to ≥ 3,200 RGCs/mm² (red). Neighbour maps illustrate the number of neighboring cells around a given cell within a radius of 0.165 mm, with a color scale from 0-2 (purple) to ≥ 21 neighbors (dark red).

### 2.9 Flow cytometry

Retinas freshly dissected post-euthanasia were collected in neurobasal medium (Thermo Fisher Scientific), supplemented with 10% fetal bovine serum (Thermo Fisher Scientific), 2% B-27 (Thermo Fisher Scientific), and 1% L-glutamine (Merck Life Science). The dissected retinas were mechanically processed with a scalpel. Following gentle resuspension through pipetting for enhanced cell dissociation, 0.2% collagenase A (0.223 U/mg; Roche Diagnostics GmbH, Mannheim, Germany) in Dulbecco’s-modified Eagle’s medium (DMEM) was added and incubated for 30 minutes at 37 °C. Subsequently, cellular suspensions were filtered through a 70-μm CorningTM cell strainer (Thermo Fisher Scientific) and promptly centrifuged for 5 minutes at 600g. The resulting cell pellets were resuspended in complete DMEM medium, and primary fluorescence-labeled antibodies (1:250 anti-mouse CD11b-FITC and 1:500 anti-mouse CD45-PE; eBioscience, Thermo Fisher Scientific) were added. After a 30-minute incubation at 4 °C, two washing steps were performed, and the cells were ultimately analyzed using a FACS Canto flow cytometer (Becton Dickinson, Franklin Lakes, NJ, United States). Flow cytometry data were analyzed with FlowJo software (FlowJo LLC, Ashland, OR, United States) at the Tissue Culture Facility (ACTI, University of Murcia and IMIB).

### 2.10 Plasma obtention, retinal protein extraction, and ELISA assays

Six, 24 or 72h after LPS administration, blood was collected from the heart of pentobarbital overdosed mice, mixed with citrate buffer (3.3% in double distilled water) and placed in ice for 15 min. Then, samples were centrifuged at 3,000 rpm for 10 min at 4°C, the plasma collected and immediately frozen at -80°C until analysis.

Right after blood extraction, retinas from the same animals were fresh dissected and immediately submerged in Pro-Prep™ (Intron Biotechnology, Seongram, South Korea) and dissociated with a hand shredder. After 1h, samples were centrifuged for 15 min at 13,000 rpm. Finally, supernatants were collected and stored at -80°C until analysis. Controls were samples from intact animals.

Each cytokine was measured individually using murine TNF, IFN-γ and IL1-β ELISA kits from Raybiotech (ELM-TNFa-CL-1, ELM-IFN-g-CL-1 and ELM-IL1-b-CL-1, respectively, Bionova Científica, Madrid, Spain), and LT-α from Cloud Clone (SEA134Mu-96T; Bionova Científica, Madrid Spain) following the manufacturer´s instructions. Absorbances were measured at 450 nm in a spectrophotometer and concentrations calculated from standard curves.

### 2.11 Statistical analyses

The data were analyzed and graphed using GraphPad Prism v.7 (GraphPad Software, San Diego, CA, USA), and the results are presented as mean ± standard deviation (SD). Significance was determined at *p*<0.05. Detailed information regarding the statistical tests employed can be found in the Results section.

## 3 Results

### 3.1 LPS dose determination

The response to LPS is modulated by the sex, age and strain of the mouse, as well as environmental factors such as diet (18, 35, 52). Therefore, the concentration of LPS used varies widely between reports, even within the same mouse strain. We started by determining LPS dose in C57BL/6J male mice from our facility (CEIB, IMIB, Murcia Spain). In the literature it has been described that males are more sensitive to LPS administration, therefore, we decided to test the appropriate LPS dose in males (30, 36). At first, we looked at whether there was RGC loss 3 days after LPS administration. (**Supplementary Figures 1A, B**). The lowest concentrations, 2 to 4 mg/kg, resulted in a loss of 10% of RGCs, while 5 and 7 mg/kg concentrations caused a 20% loss. A 10 mg/kg dose was lethal for these mice. At 3 days, there were no differences on RGC survival between 5 and 7 mg/kg. Finally, we compared the effect of the two doses at day 7 to confirm that RGC viability was still the same (**Supplementary Figure 1C**). As there were no differences in RGC survival between the two doses, all experiments were performed at the concentration of 5 mg/kg, being less toxic to mice (**Supplementary Figure 1D**).

### 3.2 Differential susceptibility of vision forming and non-vision forming RGCs to systemic inflammation

We then analysed the time course of the loss of Brn3a-expressing RGCs (vision-forming RGCs; Brn3a^+^RGCs) and melanopsin-expressing RGCs (non-vision-forming RGCs, M1 to M3 subtypes of intrinsically photosensitive RGCs; m^+^RGCs) (39) after LPS administration.

**Figure 2:**
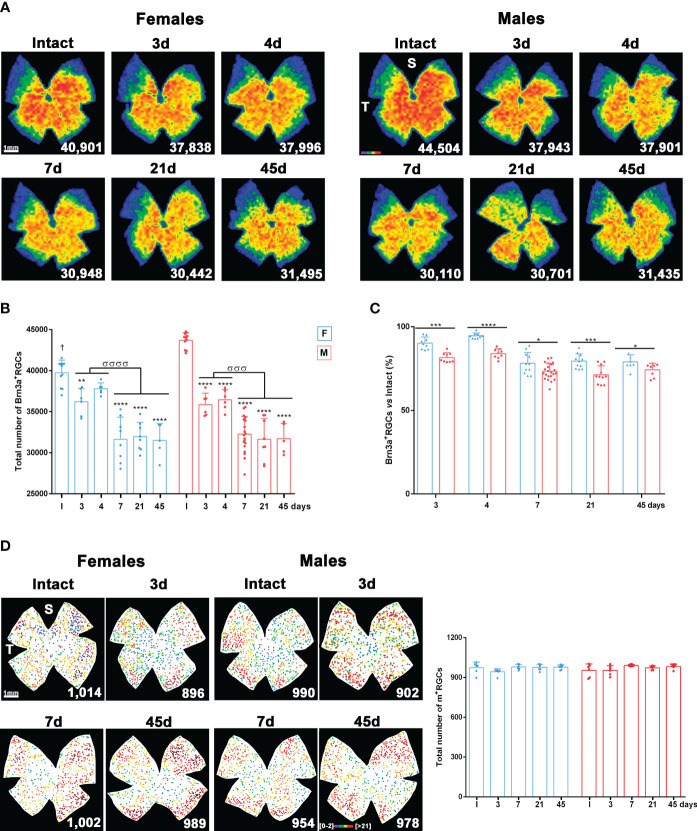
Systemic inflammation causes loss of vision forming RGCs but not of non-vision forming RGCs. **(A)** Isodensity maps showing the homogeneous loss of Brn3a^+^RGCs in female and male mice from 3 to 45 days after intraperitoneal administration of 5 mg/kg of LPS compared to intact retinas. These maps show the density of Brn3a^+^RGCs with a colour scale that goes from 0-500 (purple) to ≥ 3,200 RGCs/mm^2^ (red). Below each map is shown the number of Brn3a^+^RGCs quantified in the original retina. **(B)** Column graph showing the mean total number ± SD of Brn3a^+^RGCs in female (blue columns) and male (red columns) intact retinas and retinas analysed from 3 to 45 days after intraperitoneal administration of LPS. *Significant loss *vs*. intact (**p<0.01; ***p<0.001; ****p<0.0001). ^σ^Significant loss between time points (^σσσ^ *p*<0.001; ^σσσσ^ *p*<0.0001). One-way ANOVA within sexes, *post-hoc* Tukey’s test. †Females have significantly lower number of Brn3a^+^RGCs than males (Unpaired T-test; *p*<0.0001). **(C)** Column graph showing the mean percentage ± SD of Brn3a^+^RGCs loss in female (blue columns) and male (red columns) after LPS administration respect to intact retinas (100%). *Significant difference between females and males (*p<0.05; ***p<0.001; ****p<0.0001. Two-way ANOVA Šidák’s multiple comparison test (time *p*<0.0001; sex *p*<0.0001). **(D)** Left: Neighbour maps depicting the distribution of m^+^RGCs in intact male and female retinas and retinas analysed from 1 to 45 days after LPS administration. Neighbour maps show the number of neighbour m^+^RGCs around a given m^+^RGC in a radius of 0.165 mm with a colour scale that goes from 0-2 (purple) to ≥ 21 neighbours (dark red). Below each map the number of m^+^RGCs counted in that retina is shown. Right, column graph showing the mean total number ± SD of m^+^RGCs in the same groups. S: superior pole. T: temporal pole. F: females. M: males.

In both sexes, there was a significant and diffuse loss of Brn3a^+^RGCs on day 3. This loss continued until day 7, when it stabilised (**Figures 2A, B**). Females (blue bars) had significantly fewer Brn3a^+^RGCs than males (red bars). Therefore, to compare RGC loss between sexes, we calculated the percentage of RGC survival compared with intact retinas. As shown in **Figure 2C**, Brn3a^+^RGC loss was at all times proportionally higher in males than in females (from day 7, 27 ± 0.4% loss in males, 20 ± 0.6% in females).

The population of m^+^RGCs did not differ between males and females. In addition, unlike Brn3a^+^RGCs, they were resistant to LPS-induced systemic inflammation (**Figure 2D**).

### 3.3 Systemic inflammation causes retinal swelling

Retinas of females and males were imaged longitudinally with SD-OCT before and after LPS administration.

**Figure 3:**
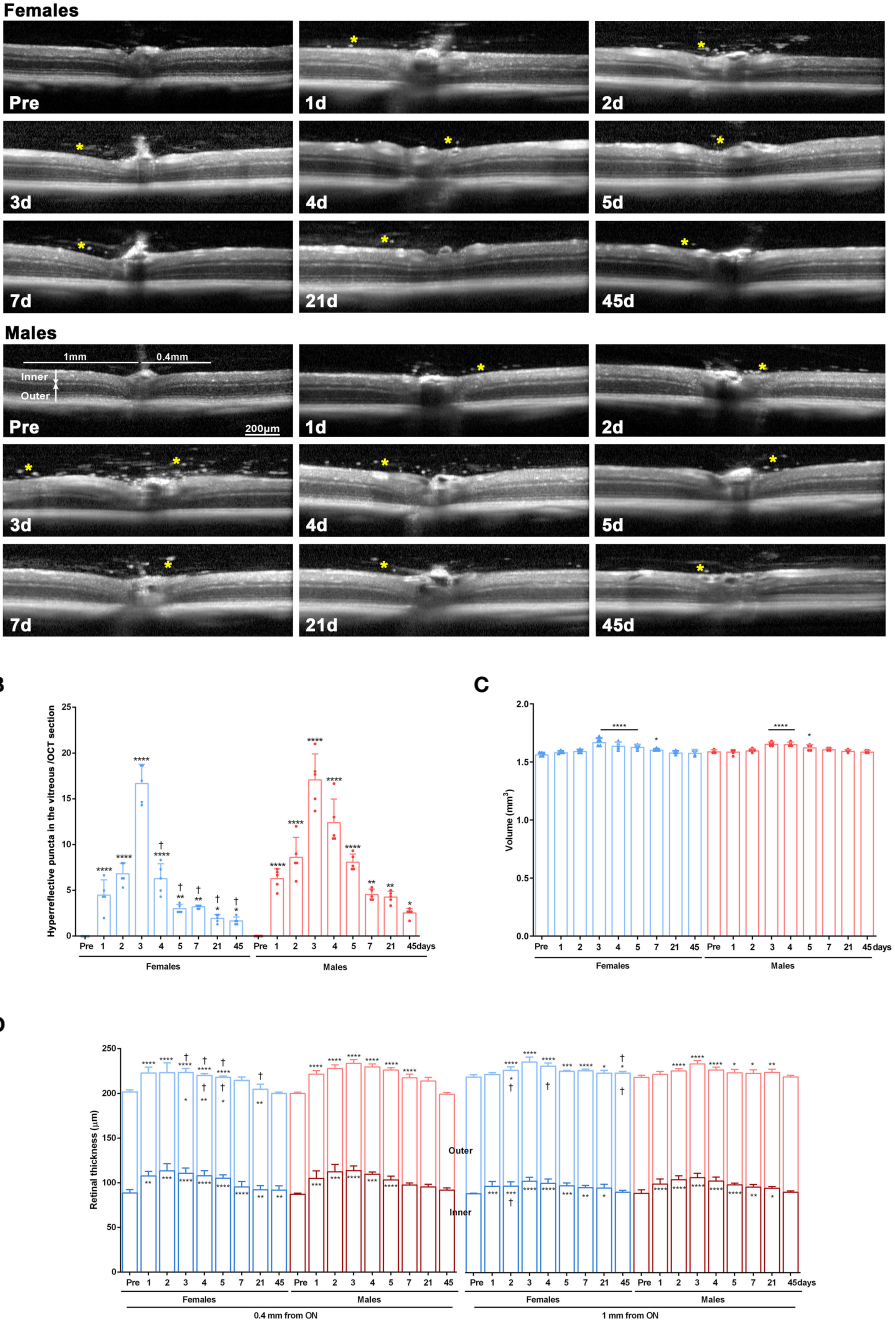
Retinal swelling after systemic LPS administration. **(A)** OCT sections spanning the optic disk acquired from female and male mice before LPS administration (pre) and from 1 to 45 days after LPS administration. Yellow asterisks mark hyperreflective puncta in the vitreous. **(B)** Column graph showing the mean number ± SD of hyperreflective puncta/OCT section. *vs. baseline values (pre) (*p<0.05; ***p<0.01; ****p<0.0001). †Females *vs.* males at the same time points (*p*<0.01). Two-way ANOVA Šidák’s multiple comparison test. **(C)** Column graph showing the mean retinal volume (µm^3^) ± SD measured from the OCT images. *Significant *vs*. baseline values (pre) (*p<0.05; ****p<0.0001. Two-way ANOVA Šidák’s multiple comparison test, time *p*<0.0001; sex *p*>0.05). **(D)** Column graph showing the mean thickness (µm) ± SD of the inner, outer, and total retina measured from the OCT images at 0.4 mm and 1 mm from the optic disc. Symbols inside the columns: statistical differences in the thickness of the inner or outer retina. Symbols above columns: differences in total thickness. *vs. baseline values (pre) (*p<0.05; **p<0.01; ***p<0.001; ****p<0.0001). †Females *vs.* males at the same time points (*p*<0.01). Two-way ANOVA Šidák’s multiple comparison test (for 0.4 mm inner: time *p*<0.0001; sex *p*>0.05; for 0.4 mm outer: time *p*<0.0001; sex *p*<0.05; for 0.4 mm total: time *p*<0.0001; sex *p*<0.0001; for 1 mm inner: time *p*<0.0001; sex *p*<0.05; for 1 mm outer: time *p*<0.001; sex *p*<0.001; for 1 mm total: time *p*<0.0001; sex *p*<0.001).

In the baseline images, the vitreous was clear. However, after LPS administration, hyperreflective puncta, presumably infiltrated cells, were visible in the vitreous from day 1 to day 45 in both sexes. Their numbers were significantly higher than at baseline imaging at all time points and were significantly more abundant in males than females from day 4 onwards (**Figures 3A, B**).

The retinal volume increased early after LPS administration similarly in both sexes (**Figure 3C**). Next, we measured the inner, outer and total retinal thickness at 0.4 mm and 1 mm from the optic disc (**Figure 3D**) and observed a significant thickening of the total retina, mainly due to enlargement of the inner retina. These changes were more marked in females than in males except at day 2 in the inner retina at 1mm.

### 3.4 Microglial activation and CD45^+^ CD11b^-^ cell recruitment

**Figure 4:**
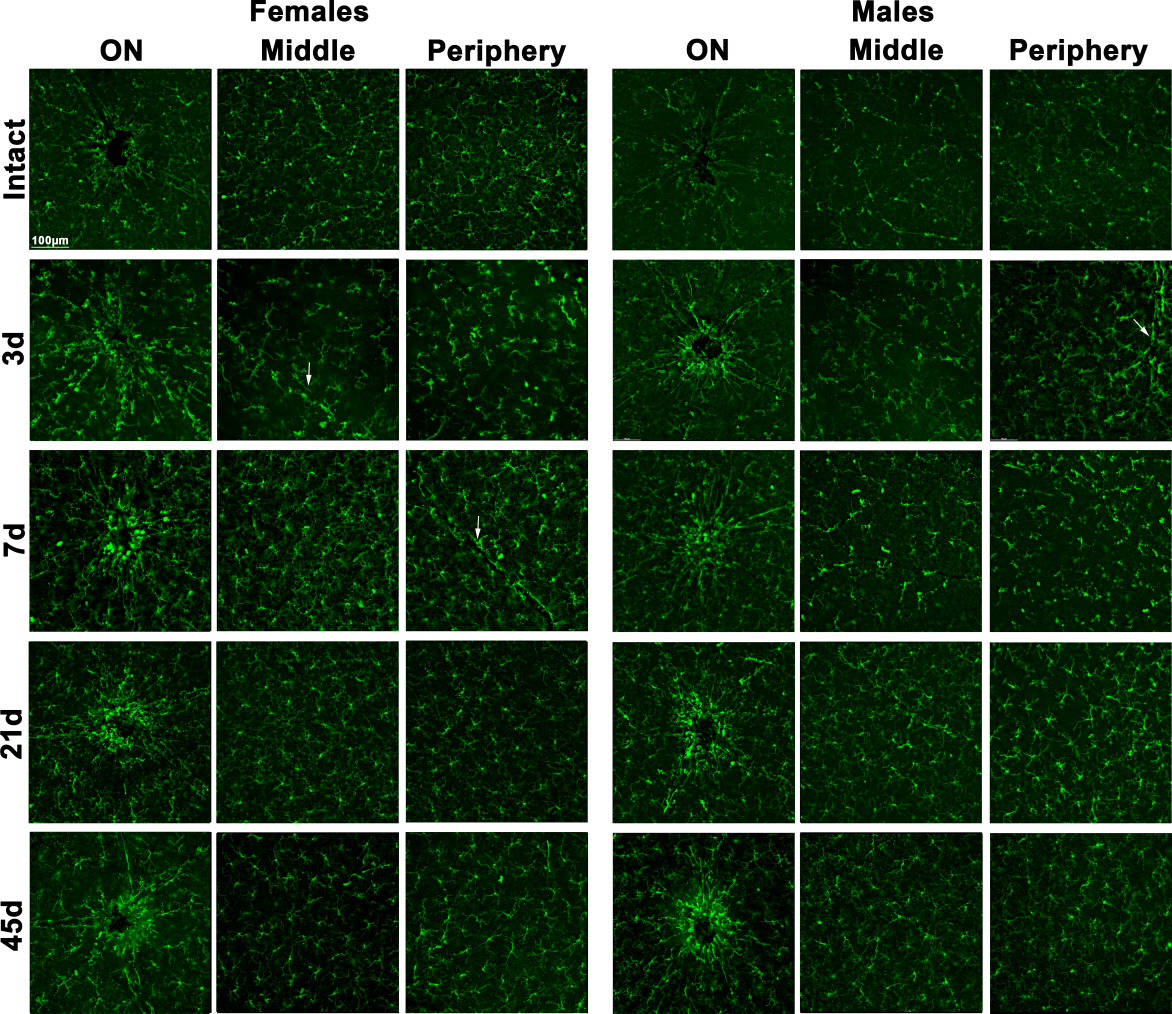
Microglial morphological activation in the ganglion cell layer after systemic inflammation. Magnifications taken from the optic nerve head (ON), centre and periphery of flat-mounted retinas of intact male and female mice, and retinas analysed from 3 to 45 days after LPS administration, showing Iba1^+^ cells (microglia or infiltrated macrophages). White arrows point to retinal vessels.

In flat-mounts, morphological activation of Iba1^+^microglia/macrophages was observed as soon as day 3 after LPS administration, and started to resume at 21 days, though still some microglial cells showed signs of activation, mostly around the optic nerve until day 45 (**Figure 4**, higher magnifications are shown in **Supplementary Figure 1E**). Iba1^+^cells were hypertrophic, with swollen cell bodies and few ramifications. In addition, amoeboid cells were found around retinal vessels, probably microglial cells, perivascular macrophages or infiltrating monocytes.

**Figure 5:**
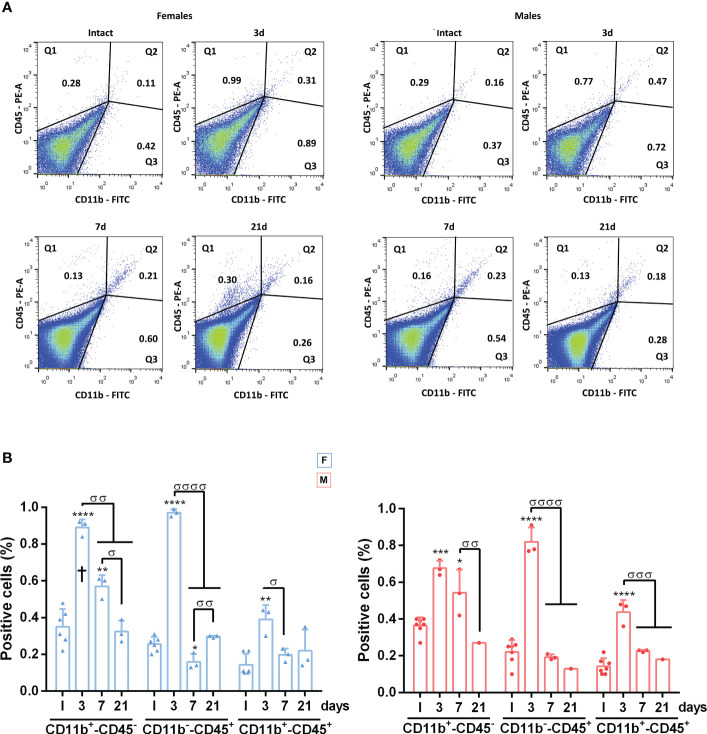
Dynamics of CD45^+^CD11b^+^, CD45^+^CD11b^-^ and CD45^-^CD11b^+^ cells in the retina after systemic inflammation. **(A)** Representative flow cytometry dot plots showing the percent of CD45^+^/CD11b^+^, CD45^+^CD11b^-^ and CD45^-^CD11b^+^ cells in intact male and female mice retinas, and retinas analysed at 3, 7 and 21 days after LPS administration. **(B)** Flow cytometry quantification graphs showing the percent ± SD of CD45^-^CD11b^+^, CD45^+^CD11b^-^ and CD45^+^/CD11b^+^ cells. *Significant *vs*. intact (*p<0.05; **p<0.01; ***p<0.001; ****p<0.0001). ^σ^Significance between different time points (^σσ^ *p*<0.01; ^σσσ^ *p*<0.001; ^σσσσ^ *p*<0.0001). † Females *vs.* males at the same time point (*p*<0.05). Two-way ANOVA Šidák’s multiple comparison test (for all cell typestime *p*<0.0001; sex *p*>0.05). F: females. M: males.

Because retinal swelling could be related to cell infiltration in the retina as well as to microglial activation, we quantified the proportion of CD11b^+^ (myeloid cells: microglial cells or macrophages, irrespective of their state of activation) or CD45^+^ cells (broad leukocyte marker: expressed by activated microglia/macrophages and by monocytes or lymphocytes [reviewed in (53)] by flow cytometry. In both sexes, there was a significant increase in CD11b^+^ and CD45^+^ cells at 3 and 7 days (**Supplementary Figure 2**) returning to basal levels on day 21, in agreement with the morphological activation. When cell populations were separated according to their marker combination, we observed an increase in steady-state microglia/macrophages (CD11b^+^CD45^-^) at 3 and 7 days, which was significantly higher in females on day 3. In both sexes, there was a similar transient increase in infiltrating neutrophils, monocytes or lymphocytes (CD11b^-^CD45^+^) at 3 days and a smaller but still significant increase in activated microglia/macrophages (CD11b^+^CD45^+^) (**Figure 5**).

### 3.5 Proinflammatory cytokine profile in retinas and plasma

**Figure 6:**
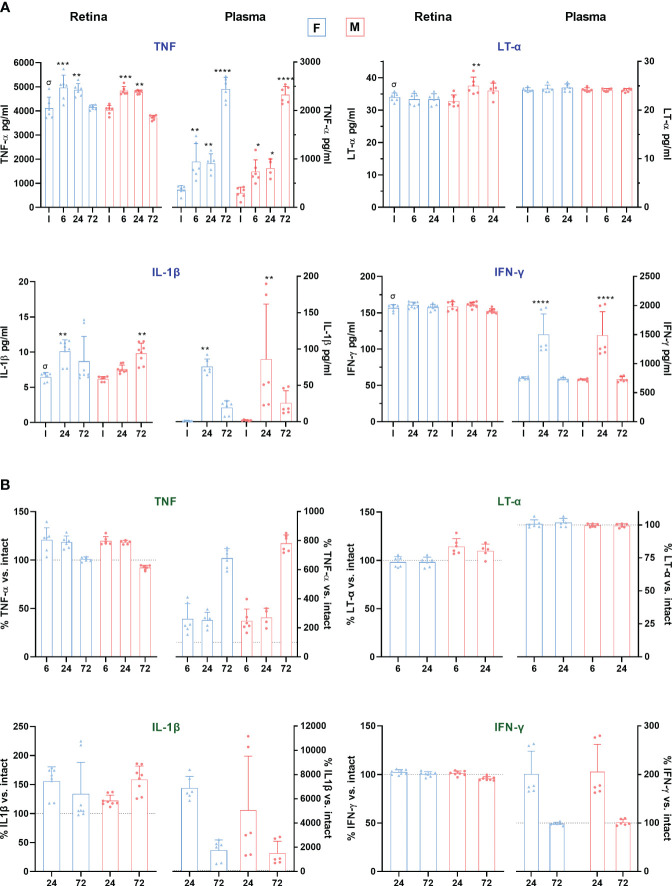
Proinflammatory cytokine levels in retina and plasma after systemic inflammation. **(A)** Column graphs showing the mean ± SD concentration (pg/mL) of TNF, LT-α, IL-1β and IFN-γ, in plasma and retinas from intact male and female mice, and retinas analysed from 6 to 72 h after LPS administration. Plasma and retinal extracts are animal matched. *Significantly different *vs.* intact (**p<0.01; ***p<0.001; ****p<0.0001); †*p*<0.01 females *vs.* males at the same time point and treatment. Two-way ANOVA Šidák’s multiple comparison test (treatment *p*<0.0001; sex *p*>0.05). *^σ^p*<0.01 plasma *vs*. retina in intact animals (Mann Whitney Test). **(B)** Column graphs showing the averaged percentage ± SD of TNF, LT-α, IL-1β and IFN-γ compared to intact (100%) in the same groups as above. The dotted line marks 100%. In all graphs, the range of the Y-axis has been adjusted for each cytokine and sample. F: females. M: males.

We measured pro-inflammatory cytokine levels by ELISA in matched retinal and plasma samples at early time points after LPS administration (**Figure 6A**). TNF levels increased significantly in the retinas of both sexes 6 and 24h after LPS exposure. In plasma, this increase was observed at 6h and progressively increased, especially at 72h. Lymphotoxin-α (LT-α), the non-canonical ligand of TNFR1, whose canonical ligand is TNF, was increased only in male retinas 6h after LPS administration. IL1-β, secreted by macrophages through inflammasome activation after LPS challenge (54), was significantly upregulated in the plasma of females and males at 24h, while this increase was observed in the retina at 24h in females and 72h in males.

IFN-γ is necessary for LPS-responsive gene induction and facilitates the production of several proinflammatory mediators (55). In our mice model, IFN-γ levels in plasma increased in both sexes after 24 h, without changes in the retina. Contrary to the other cytokines measured, IFN-γ basal levels were higher in plasma than in retina [4.6-fold]. Indeed, and surprisingly, basal retinal levels of TNF, IL-1β and LT-α were significantly higher in retina than in plasma, exceeding plasma levels by a factor of about 12, 4 and 1.4, respectively. Yet, the relative increment in TNF and IL-1β was much higher in plasma than in the retina (**Figure 6B**). By contrast, LT-α was not altered in plasma.

### 3.6 RGC neuroprotection by P2X7R and TNFR1 antagonists

In view of the above, we decided to block, alone or in combination, the P2X7 receptor (P2X7R), which induces IL-1β secretion by inflammasome activation, and the TNF receptor 1 (TNFR1), which activates the extrinsic pathway of apoptosis. Antagonists (α) were injected intraperitoneally (αTNFR1) or subcutaneously (αP2X7R) on a daily basis from day 1 after LPS administration.

The dose of the αTNFR1 (R7050) was already established in mice (43). For the αP2X7R (ITH15004), we did a preliminary experiment on LPS-treated male mice using a dose of 15 mg/kg based on *in vitro* assays (**Supplementary Figure 3**). This dose is comparable to those used for evaluating the effect of ITH15004 on the LPS-induced IL-1β release in ATP-stimulated murine peritoneal macrophages, as an eligible model of inflammation. In those experiments, the selective αP2X7R halved the IL-1β release from 1 µM (44). In our experiments, there was significant RGC rescue at this dose. To determine the effect of higher concentrations, we tried 30 and 60 mg/kg. These two higher doses did not improve RGC neuroprotection, so we performed all subsequent experiments at the lowest dose of 15 mg/kg.

**Figure 7:**
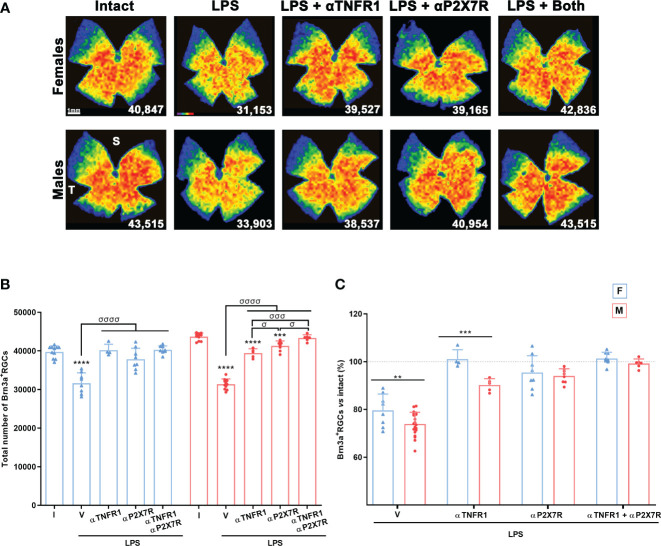
Antagonism of TNFR1 and P2X7R rescues RGCs from systemic inflammation. **(A)** Isodensity maps showing the distribution of Brn3a^+^RGCs in retinas of intact male mice and mice treated with LPS+vehicle, LPS and TNFR1 antagonist (αTNFR1), LPS and P2X7R antagonist (αP2X7R), and LPS and αP2X7R + αTNFR1. Retinas were analysed 7 days after the injection of LPS. **(B)** Column graph showing the mean total number ± SD of Brn3a^+^RGCs the same groups. *Significant *vs.* intact (***p<0.001; ****p<0.0001); ^σ^Significant between groups (^σ^ *p*<0.05; ^σσσ^ *p*<0.001; ^σσσσ^ *p*<0.0001). One-way ANOVA within sexes, *post-hoc* Tukey’s test. **(C)** Column graph showing the averaged percentage ± SD of Brn3a^+^RGCs in the same groups as before with respect to intact retinas (100%). *Significant differences between females and males (**p<0.01; ***p<0.001; Two-way ANOVA Šidák’s multiple comparison test (treatment *p*<0.0001; sex *p*<0.0001). F: females, M: males. I: intact, V: vehicle.

Each antagonist was able to reverse LPS-induced Brn3a^+^RGC loss. Both antagonists worked better in females, with no differences compared to intact animals, while RGC neuroprotection was not complete in males. The combination of both treatments significantly improved neuroprotection in males compared with either treatment alone. (**Figures 7A, B**). Strikingly, the rescue of RGCs with the TNFR1 antagonist was significantly better in females than in males (**Figure 7C**).

### 3.7 Retinal functionality

**Figure 8:**
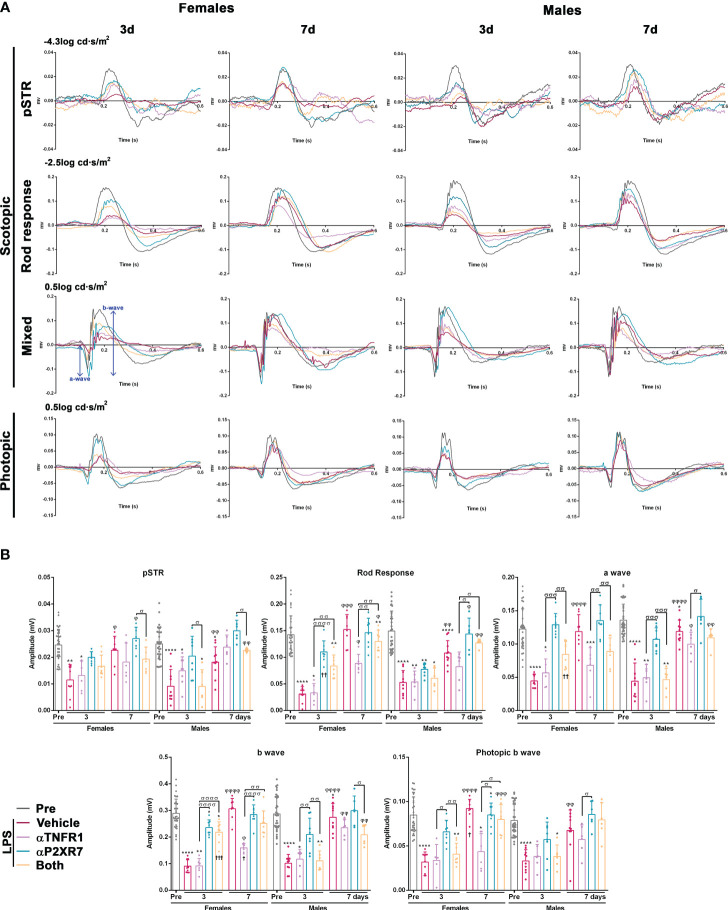
Transient impairment of retinal functionality after systemic inflammation: effect of TNFR1 and P2X7R antagonism. **(A)** Electroretinographic waves from female and male mice recorded before (PRE) and 3 and 7 days after being treated with LPS + vehicle, LPS and TNFR1 antagonist (αTNFR1), LPS and P2X7R antagonist (αP2X7R), and LPS and αP2X7R + αTNFR1. **(B)** ERG quantification bar graphs showing the mean wave amplitude (µV ± SD). Control amplitudes are baseline recordings (pre). *vs. baseline values (*p<0.05; **p<0.01***; *p*<0.001; ****p<0.0001); ^φ^3^rd^ *vs.* 7^th^ day within the same group (^φφφ^ *p*<0.001; ^φφφφ^ *p*<0.0001). ^σ^Between different groups (^σ^ *p*<0.05; ^σσ^ *p*<0.01; ^σσσ^ *p*<0.001; ^σσσσ^ *p*<0.0001). †*p*<0.001 females *vs.* males at the same time point and treatment. Two-way ANOVA Šidák’s multiple comparison test (treatment *p*<0.0001; sex *p*>0.05).

Electroretinographic waves were recorded in all animals before (pre) and at 3 and 7 days after LPS administration, with or without pharmacological treatments (**Figure 8**). LPS caused a transient decrease in all wave amplitudes at day 3 in both sexes, indicating functional impairment of the inner and outer retina. In females, all waves were completely recovered by day 7, whereas in males this recovery was incomplete except for the photopic wave. P2X7R antagonism completely restored all waves at day 3, except the b-wave in females, whereas TNFR1 antagonism restored only the photopic wave in males. The combined treatment was better than the TNFR1 antagonism alone, but not as good as the single P2X7R antagonism. There were some subtle differences between females and males, such as smaller recovery at day 3 of the a- and b- mixed waves in males than in females when treated with both antagonists, or of the rod response after P2X7R antagonism.

Finally, when we measured the implicit time, we observed that the response of the female retina was significantly slower than that of the male retina for all waves 3 days after LPS administration (**Supplementary Figure 4**). In animals treated with the antagonists, where the implicit times of females were faster, this difference was not observed.

## 4 Discussion

We show here that systemic inflammation induced by intraperitoneal administration of LPS causes transient functional impairment, swelling, microglial activation and increase in number, CD45^+^CD11b^-^ cell infiltration, vision-forming RGC death and a pro-inflammatory profile in the mouse retina. RGC death and retinal function are restored by antagonising P2X7R and TNFR1 alone or in combination, with better results for RGC survival when both receptors are targeted. Importantly, some of these events differ between male and female mice.

In our mice, systemic inflammation leads to RGC loss, which is significantly lower in females than in males and it affects only vision-forming RGCs. Non-vision-forming RGCs are more resistant than vision-forming RGCs (41, 42); their resistance may be due to protection against neuroinflammation. In fact, after optic nerve axotomy, m^+^RGCs die as Brn3a^+^RGCs during the fast phase of death, but they survive the second phase, which is secondary to the axotomy itself (41). During this second phase, Brn3a^+^RGC death is very slow but sustained, and microglial cells remain activated (56). In accordance, we show here that m^+^RGCs are not affected by the neuroinflammation triggered by LPS.

LPS rapidly induced a retinal and systemic proinflammatory status, as evidenced by increased levels of TNF and IL-1β in both tissues, LT-α only in the male retina, and IFN-γ only in plasma. Interestingly, basal levels of all these proinflammatory mediators, except IFN-γ, were significantly higher in the retina than in plasma, although their increased levels after LPS challenge were much higher in plasma, as expected. We do not know the biological significance of this, but it would be interesting to measure the basal levels of these cytokines in other areas of the CNS to determine whether their constitutive levels are higher in the CNS than in plasma or other peripheral tissues.

We found that systemic inflammation kills RGCs through both the extrinsic apoptotic pathway and the NLRP3 inflammasome pathway. This is supported by the increased levels of TNF and IL-1β in the retina and by the fact that the antagonism of the key receptors of each pathway, TNFR1 and P2X7R, rescues RGCs and, for P2X7R antagonism, retinal function.

Importantly, RGC neuroprotection in females is complete with all treatments, whereas this level of protection in males is only achieved with administration of both antagonists.

RGCs express TNFR1 (43) and can therefore be killed directly by the increased retinal levels of TNF and LT-α (in males). LT-α is a non-canonical ligand of TNFR1 and, like TNF, it also binds to other death receptors (57). This cytokine is involved in apoptosis, necroptosis and inflammation (58). The significant increase in retinal LT-α only in males may explain their higher RGC loss. LPS induces LT-α secretion by microglial cells (59) and our data indicate that this secretion is sexually dimorphic.

Activation of the NLRP3 inflammasome by P2X7R induces Gasdermin D pore formation and the release of IL-1β, leading to inflammation and pyroptosis (60). RGCs also express P2X7R (61), therefore they are vulnerable to pyroptosis. In addition, because microglial cells become neurotoxic after P2X7R activation (62–64), it is plausible that, in this model, where the number of microglial cells increases and they are activated, both mechanisms, *i.e.* direct pyroptotic RGC death and microglial neurotoxicity, take place.

OCT analysis showed retinal swelling and the appearance of hyperreflective puncta, most likely infiltrated cells, in the vitreous which were more abundant in males than in females. Further cytometric analysis showed an increase in microglial cells/macrophages in different activation states (CD45^+^CD11b^+^ and CD45^-^CD11b^+^ cells). There was also an increase in CD45^+^CD11b^-^ cells, which are probably infiltrating lymphocytes as they are not macrophages or microglial cells. Retinal swelling may be a consequence of this infiltration, the higher number of microglial cells and the subsequent inflammation. Morphological microglial activation is observed from day 3, more pronounced around the optic nerve disc and retinal vessels in agreement with (64). This geographical pattern may indicate the route of infiltration.

Early after LPS administration, inner and outer retinal function was impaired and recovered for all waves at 7 days, better in females than in males. The loss of function may be a response to the systemic shock and the early increase in pro-inflammatory mediators in both retina and plasma. Activated microglial cells in sepsis release IL-1β in microvesicles, which can potentially cause synaptic damage (54). Thus, overexpression of IL-1β in the retina may explain the early loss of function that we observe here, and its rescue in P2X7R antagonised groups. The idea that the inflammatory environment underlies the loss of retinal function is also supported by the fact that TNFR1 antagonism, while rescuing RGCs, does not restore retinal function. Surprisingly, the pSTR wave, corresponding to RGCs, also recovered despite a 20-27% loss of RGCs. The electroretinogram shows functional changes with ~50% neuronal loss or impairment (45, 65). Loss of function, as measured by the ERG, is observed when ~50% of neurons are lost or functionally impaired. Since LPS causes the loss of 20-27% of RGCs, it is possible that the pSTR decrease at early time points is due to extensive RGC dysfunction as well as loss. Later, the surviving RGCs function well, and with 73-80% still alive, the ERG is not sensitive enough to detect functional deficits. Additionally, the pSTR recovery could also be related to functional compensatory mechanisms. The transient loss of outer retinal function suggests that bipolar cells or photoreceptors (66) are also affected. Whether this loss of function is associated with death below ERG sensitivity remains unclear and requires further investigation.

## 5 Conclusions

The most important conclusion to be drawn from our work is that the response of the retina to systemic LPS is sexually dimorphic. Females show a better functional recovery, less cell infiltration in the vitreous, less loss of vision-forming RGCs and a better response to treatment than males. Furthermore, males show increased levels of retinal LT-α, which is not observed in females. This sexual dimorphism may be related to estrogen receptors, which have been shown to inhibit proinflammatory cytokines (67), highlighting the need for preclinical studies in both sexes in diseases with an inflammatory component, either systemic or neurodegenerative, such as Parkinson’s disease (68).

The two functional subtypes of RGCs show differential susceptibility to systemic inflammation. The intrinsic resilience of non-vision-forming RGCs to various insults is well known, a resilience that may be shared by other CNS neurons. Therefore, one of the main goals of current research is to isolate the basis of this resilience in order to identify successful neuroprotective therapies.

Finally, our data provide evidence that the NLRP3 inflammasome and the extrinsic pathway of apoptosis play a role in RGC death induced by systemic inflammation. In addition, P2X7R activation is also involved in the early loss of retinal function. Thus, this work provides the basis for further research into their possible role in retinal or CNS diseases with a systemic or local inflammatory component.

## Data availability statement

The original contributions presented in the study are included in the article/**Supplementary Material**. Further inquiries can be directed to the corresponding author.

## Ethics statement

The animal study was approved by Institutional Animal Care and Use Committee at University of Murcia [Murcia, Spain] and performed according to the guidelines of our Institution [approved protocol A13210201]. The study was conducted in accordance with the local legislation and institutional requirements.

## Author contributions

KR-R: Data curation, Formal analysis, Investigation, Methodology, Writing – review & editing. MN-M: Data curation, Formal analysis, Investigation, Methodology, Writing – review & editing. FL-R: Data curation, Formal analysis, Investigation, Methodology, Writing – review & editing. AG-O: Data curation, Formal analysis, Investigation, Methodology, Writing – review & editing. FC: Investigation, Methodology, Resources, Writing – review & editing. DG-B: Investigation, Methodology, Resources, Writing – review & editing. CM: Methodology, Writing – review & editing. CG-R: Investigation, Methodology, Writing – review & editing. CdlR: Funding acquisition, Investigation, Resources, Supervision, Writing – review & editing. MV-S: Conceptualization, Funding acquisition, Writing – review & editing. MA-B: Conceptualization, Data curation, Formal analysis, Funding acquisition, Investigation, Methodology, Project administration, Resources, Supervision, Validation, Visualization, Writing – original draft.

## Supplementary material

Supplementary Figure 1 LPS dose determination. **(A)** Isodensity maps showing the homogeneous loss of Brn3a^+^RGCs in the retinas of male mice treated with increasing intraperitoneal doses of LPS (mg/kg) compared to intact retinas. These maps show the density of Brn3a^+^RGCs with a colour scale that goes from 0-500 (purple) to ≥ 3,200 RGCs/mm^2^ (red). Below each map is shown the number of RGCs quantified in the original retina. (**B)** Column graph showing the mean total number ± standard deviation (SD) of Brn3a^+^RGCs in intact retinas and retinas analysed 3 days after intraperitoneal administration of increasing doses of LPS. **(C)** Column graph showing the mean total number ± standard deviation of Brn3a^+^RGCs in intact retinas and retinas analysed 7 days after intraperitoneal administration of 5 or 7 mg/kg of LPS. **(D)** XY graph (LPS dose *vs*. mouse survival) showing the percentage of mice surviving each of the LPS doses tested. **(E)** Magnifications taken from the optic nerve head (ON), centre and periphery of flat-mounted retinas of intact male and female mice, and retinas analysed from 3 to 45 days after LPS administration, showing Iba1^+^ cells (microglia or infiltrated macrophages).

Supplementary Figure 2 Transient increase of CD45^+^ and CD11b^+^ cells in the retina after systemic inflammation. **(A)** Representative flow cytometry dot plots showing the percent of CD45^+^ or CD11b^+^ cells in intact male and female mice retinas, and retinas analysed at 3, 7 and 21 days after LPS administration. **(B)** Flow cytometry quantification graph showing the mean percent ± SD of CD45^+^ (left) or CD11b^+^ (right) cells. *Significant compared to intact retinas (**p*<0.05; ***p*<0.01). Two-way ANOVA Šidák’s multiple comparison test (For both cell typestime *p*<0.0001; sex *p*>0.05). F: females. M: males.

Supplementary Figure 3 Neuroprotective dose of the P2X7R antagonist ITH15004. **(A)** Isodensity maps showing the distribution of Brn3a^+^RGCs in retinas of intact male mice and mice treated with LPS + vehicle, or with LPS and increasing subcutaneous doses of the P2X7R antagonist (αP2X7R) ITH15004 (mg/kg). Retinas were analysed 7 days after LPS administration. Below each map the number of RGCs quantified in the original retina is shown. **(B)** Column graph showing the mean total number ± SD of Brn3a^+^RGCs in the same groups. ^σ^Significant *vs*. vehicle (^σσσσ^ *p*<0.0001) *Significant compared to intact retinas (**p*<0.05; ***p*<0.01; ****p*<0.001; *****p*<0.0001); ^φ^15 mg/kg *vs.* 30 mg/kg (*p*<0.05). One-way ANOVA, *post-hoc* Tukey’s test.

Supplementary Figure 4 Changes in the implicit time after systemic inflammation and effect of TNFR1 and P2X7R antagonism. Colum bar graphs showing the mean implicit time (ms ± SD) of the ERG response in female and male mice recorded before (PRE) and 3 and 7 days after being treated with LPS + vehicle, LPS and TNFR1 antagonist (αTNFR1), LPS and P2X7R antagonist (αP2X7R), and LPS and αP2X7R+ αTNFR1 *vs.* baseline values (**p*<0.05; *****p*<0.0001); ^φ^3^rd^ *vs.* 7^th^ day within the same group (^φφφ^ *p*<0.001; ^φφφφ^ *p*<0.0001). †*p*<0.05 females *vs.* males at the same time point and treatment. Two-way ANOVA Šidák’s multiple comparison test (treatment *p*<0.0001; sex *p*>0.05).
