# Supplementary figures and images for: Retinal response to systemic inflammation differs between sexes and neurons
Source: Front Immunol. 2024 Feb 7;15:1340013. doi: 10.3389/fimmu.2024.1340013 (PMC10880026; doi:10.3389/fimmu.2024.1340013)

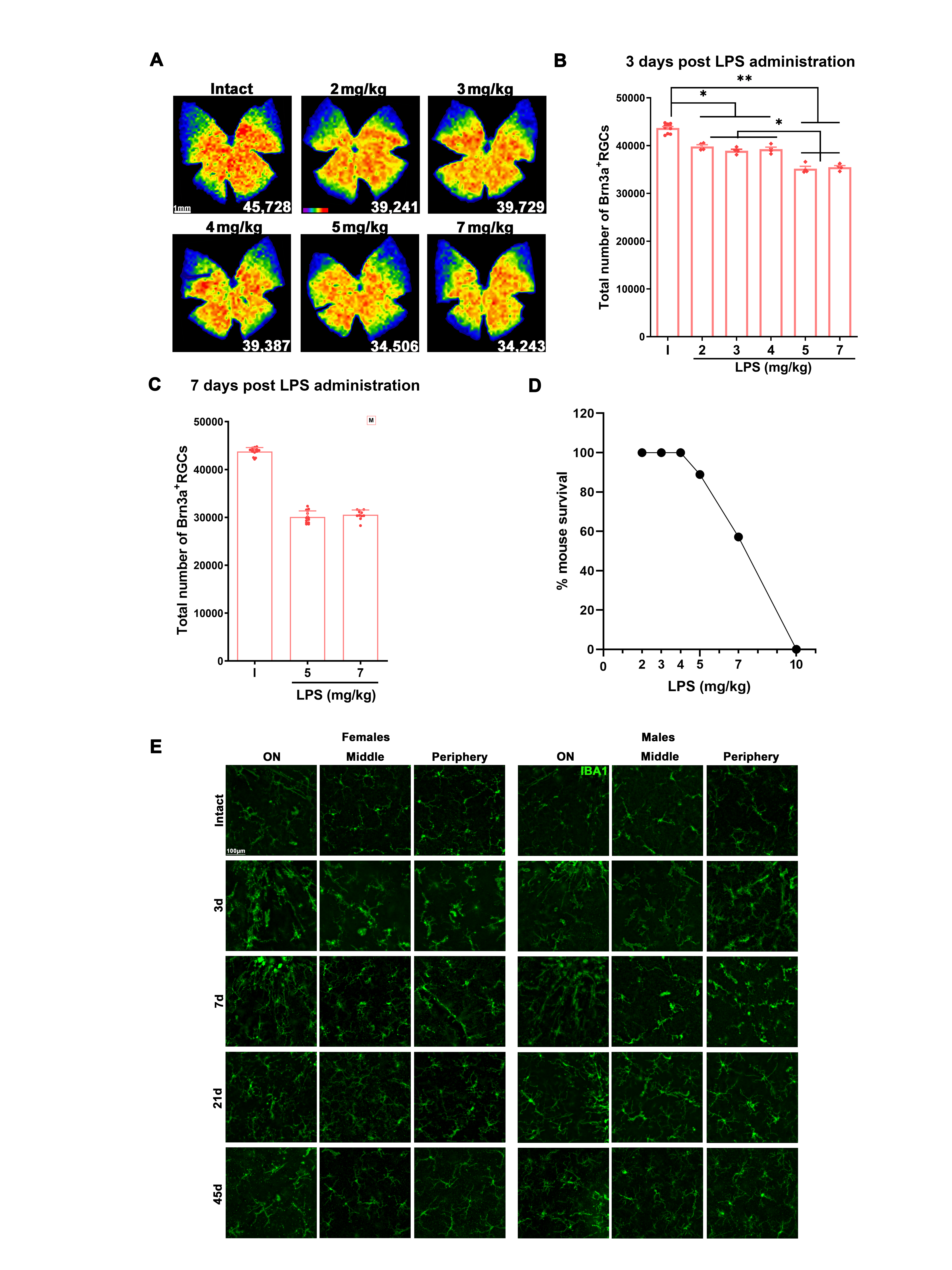

Supplement: Supplementary Figure 1 — LPS dose determination. (A) Isodensity maps showing the homogeneous loss of Brn3a+RGCs in the retinas of male mice treated with increasing intraperitoneal doses of LPS (mg/kg) compared to intact retinas. These maps show the density of Brn3a+RGCs with a colour scale that goes from 0-500 (purple) to ≥ 3,200 RGCs/mm2 (red). Below each map is shown the number of RGCs quantified in the original retina. (B) Column graph showing the mean total number ± standard deviation (SD) of Brn3a+RGCs in intact retinas and retinas analysed 3 days after intraperitoneal administration of increasing doses of LPS. (C) Column graph showing the mean total number ± standard deviation of Brn3a+RGCs in intact retinas and retinas analysed 7 days after intraperitoneal administration of 5 or 7 mg/kg of LPS. (D) XY graph (LPS dose vs. mouse survival) showing the percentage of mice surviving each of the LPS doses tested. (E) Magnifications taken from the optic nerve head (ON), centre and periphery of flat-mounted retinas of intact male and female mice, and retinas analysed from 3 to 45 days after LPS administration, showing Iba1+ cells (microglia or infiltrated macrophages). [file Image_1.tif]

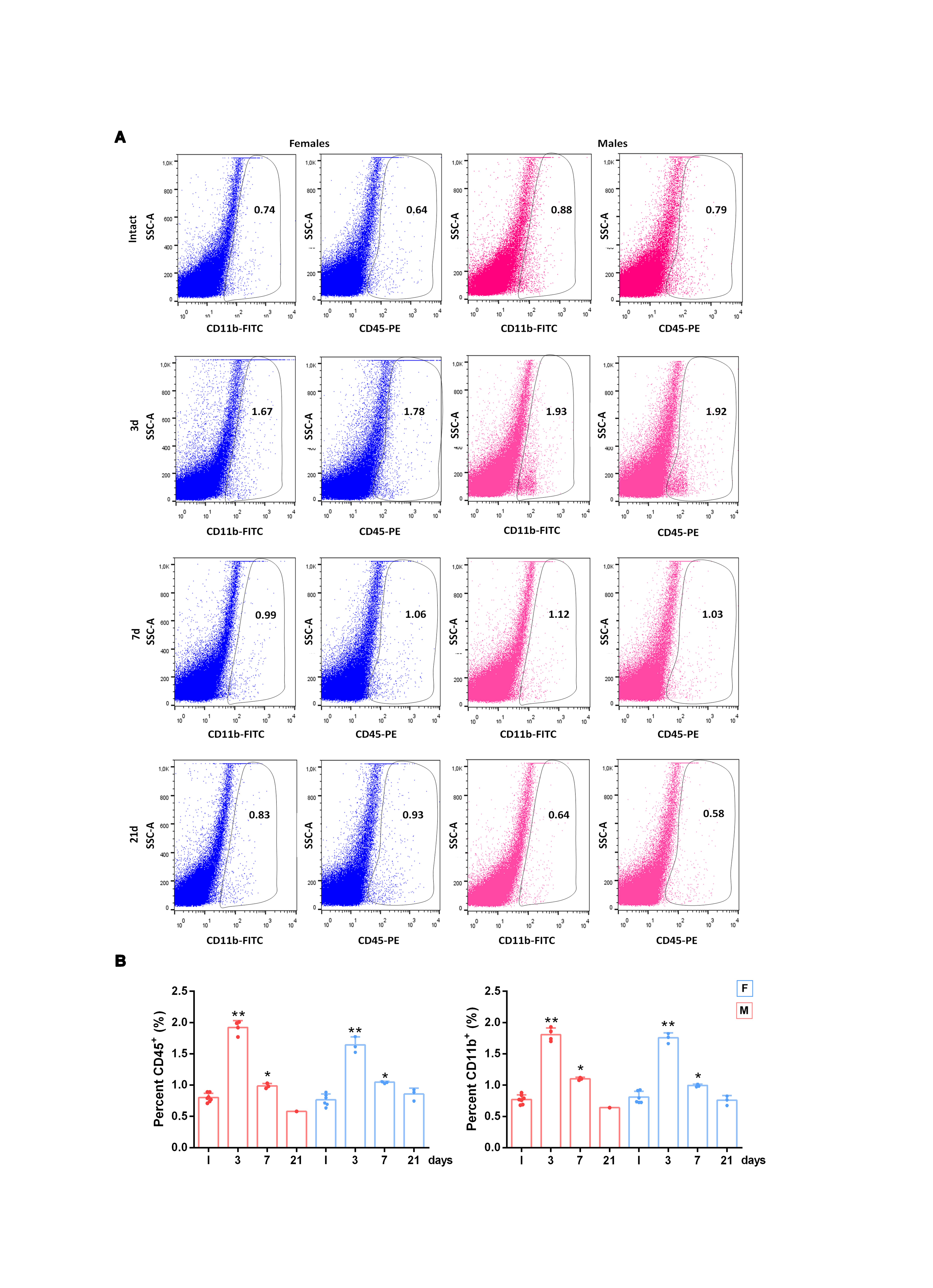

Supplement: Supplementary Figure 2 — Transient increase of CD45+ and CD11b+ cells in the retina after systemic inflammation. (A) Representative flow cytometry dot plots showing the percent of CD45+ or CD11b+ cells in intact male and female mice retinas, and retinas analysed at 3, 7 and 21 days after LPS administration. (B) Flow cytometry quantification graph showing the mean percent ± SD of CD45+ (left) or CD11b+ (right) cells. *Significant compared to intact retinas (*p<0.05; **p<0.01). Two-way ANOVA Šidák’s multiple comparison test (For both cell typestime p<0.0001; sex p>0.05). F: females. M: males. [file Image_2.tif]

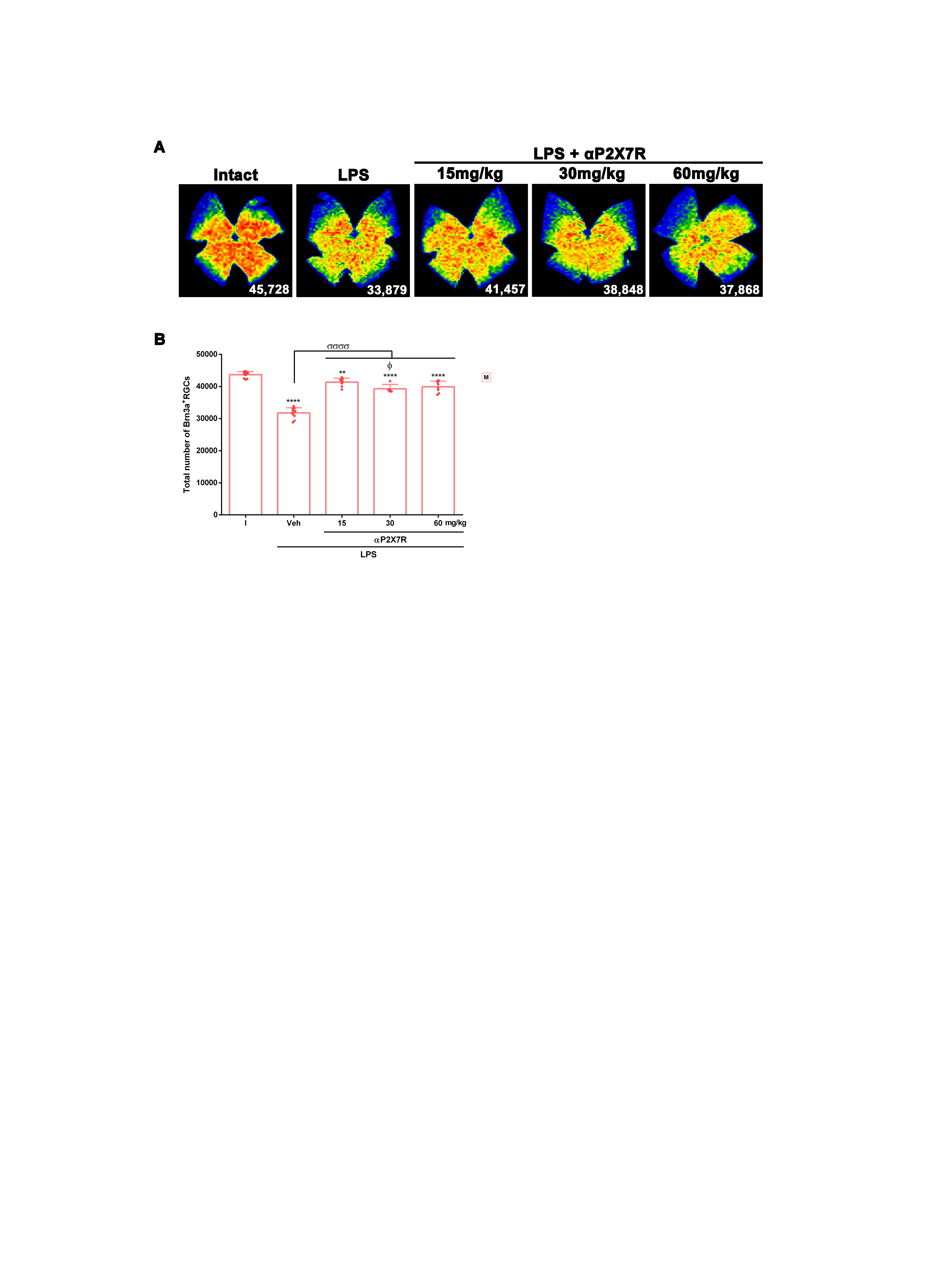

Supplement: Supplementary Figure 3 — Neuroprotective dose of the P2X7R antagonist ITH15004. (A) Isodensity maps showing the distribution of Brn3a+RGCs in retinas of intact male mice and mice treated with LPS + vehicle, or with LPS and increasing subcutaneous doses of the P2X7R antagonist (αP2X7R) ITH15004 (mg/kg). Retinas were analysed 7 days after LPS administration. Below each map the number of RGCs quantified in the original retina is shown. (B) Column graph showing the mean total number ± SD of Brn3a+RGCs in the same groups. σSignificant vs. vehicle (σσσσ p<0.0001) *Significant compared to intact retinas (*p<0.05; **p<0.01; ***p<0.001; ****p<0.0001); φ15 mg/kg vs. 30 mg/kg (p<0.05). One-way ANOVA, post-hoc Tukey’s test. [file Image_3.tif]

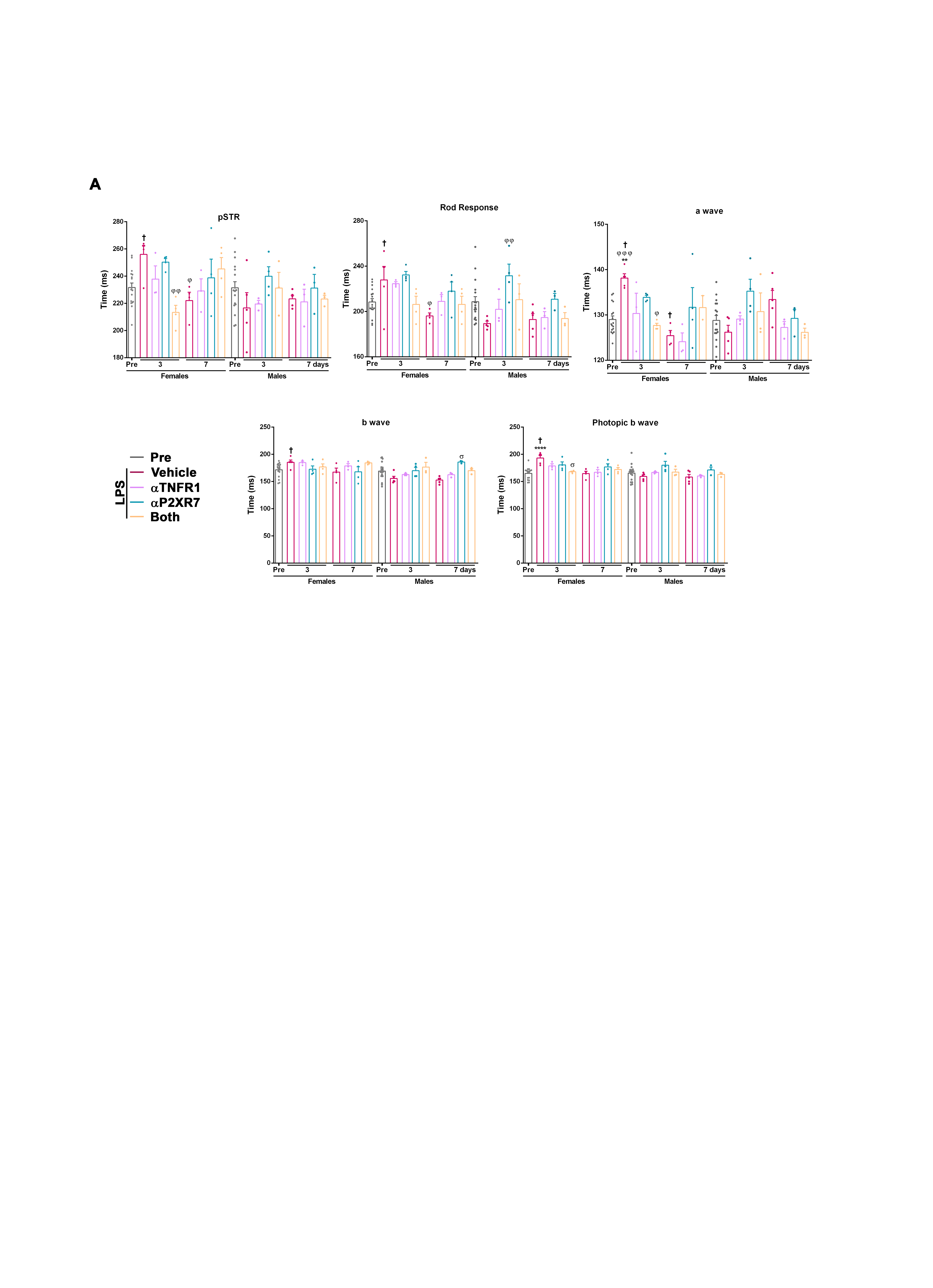

Supplement: Supplementary Figure 4 — Changes in the implicit time after systemic inflammation and effect of TNFR1 and P2X7R antagonism. Colum bar graphs showing the mean implicit time (ms ± SD) of the ERG response in female and male mice recorded before (PRE) and 3 and 7 days after being treated with LPS + vehicle, LPS and TNFR1 antagonist (αTNFR1), LPS and P2X7R antagonist (αP2X7R), and LPS and αP2X7R+ αTNFR1 vs. baseline values (*p<0.05; ****p<0.0001); φ3rd vs. 7th day within the same group (φφφ p<0.001; φφφφ p<0.0001). †p<0.05 females vs. males at the same time point and treatment. Two-way ANOVA Šidák’s multiple comparison test (treatment p<0.0001; sex p>0.05). [file Image_4.tif]
